# Persistence of SARS-CoV-2 RNA in the nasopharyngeal, blood, urine, and stool samples of patients with COVID-19: a hospital-based longitudinal study

**DOI:** 10.1186/s12985-021-01599-9

**Published:** 2021-07-01

**Authors:** Farahnaz Joukar, Tofigh Yaghubi Kalurazi, Mahmoud Khoshsorour, Sonbol Taramian, Lida Mahfoozi, Heydar Ali Balou, Alireza Jafarinezhad, Aydin Pourkazemi, Ezat Hesni, Mehrnaz Asgharnezhad, Mohammad Shenagari, Issa Jahanzad, Mohammadreza Naghipour, Saman Maroufizadeh, Fariborz Mansour-Ghanaei

**Affiliations:** 1 Gastrointestinal and Liver Diseases Research Center, GI Cancer Screening and Prevention Research Center and Caspian Digestive Diseases Research Center, Guilan University of Medical Sciences, Razi Hospital, Sardar-Jangle Ave, 41448-95655 Rasht, Iran; 2 Department of Health, Nutrition and Infectious Diseases, School of Medicine Razi Hospital, Guilan University of Medical Sciences, Rasht, Iran; 3 Gastrointestinal and Liver Diseases Research Center, Guilan University of Medical Sciences, Rasht, Iran; 4 Department of Health, Nutrition and Infectious Diseases, Guilan University of Medical Sciences, Rasht, Iran; 5 Department of Internal Medicine, School of Medicine, Gastrointestinal and Liver Disease Research Center, Guilan University of Medical Sciences, Rasht, Iran; 6 Department of Internal Medicine, School of Medicine, Razi Hospital, Guilan University of Medical Sciences, Rasht, Iran; 7 Department of Health, Nutrition and Infectious Diseases, School of Medicine, Guilan University of Medical Sciences, Rasht, Iran; 8 GI Cancer Screening and Prevention Research Center, Guilan University of Medical Sciences, Rasht, Iran; 9 Department of Microbiology, School of Medicine, Gastrointestinal and Liver Disease Research Center, Guilan University of Medical Sciences, Rasht, Iran; 10 Pars Hospital, Rasht, Iran; 11 Department of Biostatistics, School of Nursing and Midwifery, Guilan University of Medical Sciences, Rasht, Iran

**Keywords:** SARS-CoV-2, RNA, COVID-19, RT-PCR, Persistence

## Abstract

**Background:**

The persistence of severe acute respiratory syndrome-coronavirus 2 (SARS-CoV-2) RNA in the body fluids of patients with the novel coronavirus disease 2019 (COVID-19) may increase the potential risk of viral transmission. There is still uncertainty on whether the recommended quarantine duration is sufficient to reduce the risk of transmission. This study aimed to investigate the persistence of SARS-CoV-2 RNA in the nasopharyngeal, blood, urine, and stool samples of patients with COVID-19.

**Methods:**

In this hospital-based longitudinal study, 100 confirmed cases of COVID-19 were recruited between March 2020 and August 2020 in Guilan Province, north of Iran. Nasopharyngeal, blood, urine, and stool samples were obtained from each participant at the time of hospital admission, upon discharge, 1 week after discharge, and every 2 weeks until all samples were negative for SARS-CoV-2 RNA by reverse transcription-polymerase chain reaction (RT-PCR) assay. A survival analysis was also performed to identify the duration of viral persistence.

**Results:**

The median duration of viral RNA persistence in the nasopharyngeal samples was 8 days from the first positive RT-PCR result upon admission (95% CI 6.91–9.09); the maximum duration of viral shedding was 25 days from admission. Positive blood, urine, and stool RT-PCR results were detected in 24%, 7%, and 6% of the patients, respectively. The median duration of viral persistence in the blood, urine, and stool samples was 7 days (95% CI 6.07–7.93), 6 days (95% CI 4.16–8.41), and 13 days (95% CI 6.96–19.4), respectively. Also, the maximum duration of viral persistence in the blood, urine, and stool samples was 17, 11, and 42 days from admission, respectively.

**Conclusion:**

According to the present results, immediately after the hospitalized patients were discharged, no evidence of viral genetic materials was found. Therefore, appropriate treatments were selected for the patients at this hospital. However, we recommend further investigations on a larger sample size in multi-center and prospective randomized controlled trials (RCTs) to evaluate the effects of different drugs on the shedding of the virus through body secretions.

## Introduction

Coronavirus disease 2019 (COVID-19) was declared as a global pandemic by the World Health Organization (WHO) in December 2020 [1]. By November 22, 2020, this virus infected almost 57 million people around the world and caused more than 1,300,000 deaths [1]. Iran is ranked the eighth country in terms of COVID-19 mortality [2]. There were 1,189,203 confirmed cases of COVID-19 and 54,440 deaths in Iran from February 15, 2019, until December 25, 2020 [3].

The main route of COVID-19 transmission seems to be direct or indirect exposure to respiratory droplets [4, 5]. Although other routes of transmission, such as mother-to-fetus, fecal–oral, and airborne transmission, are currently controversial and subject to future investigations [5–8], epidemiological studies have shown that COVID-19 patients have had close contact with an infected individual or have been in close proximity to a patient [9].

Initially, severe acute respiratory syndrome-coronavirus 2 (SARS-Cov-2) was isolated and identified in respiratory samples by real-time reverse transcription-polymerase chain reaction (RT-PCR) assay [10]. However, in recent studies, viral nucleic acids have been also detected in urine, stool, and gastric mucosa samples [10, 11]. In a previous study, ten children with COVID-19, despite having negative nasopharyngeal RT-PCR results, still showed positive RT-PCR of throat swabs [12]. Based on the Centers for Disease Control and Prevention (CDC) guidelines, all patients with a positive respiratory RT-PCR result must be isolated for at least 10 days after symptom onset and after resolution of fever for at least 24 h [13].

Nevertheless, there are several case reports on the persistence of positive RT-PCR in patients with COVID-19, indicating the possibility of positive results after the symptoms have resolved [14, 15]. Therefore, the persistence of the virus in body fluids can increase the potential risk of viral transmission in asymptomatic or recovered patients [15]. It also remains uncertain whether the quarantine duration, recommended by the CDC, is sufficient to reduce viral transmission [16]. Since the frequency and detection time of SARS-CoV-2 RNA in body, fluids are not well understood, in this longitudinal study, we aimed to determine the persistence of SARS-CoV-2 RNA in the nasopharyngeal, blood, urine, and stool samples of patients with COVID-19, which were collected every 2 weeks by sequential sampling.

## Methods

### Study population and design

This hospital-based longitudinal study was performed between March 2020 and August 2020 during 6 months. The participants were selected by convenience sampling among hospitalized patients with a confirmed diagnosis of COVID-19 in the only referral hospital of Rasht, Guilan Province, in north of Iran. A positive case of COVID-19 was defined as a patient with a positive quantitative real-time RT-PCR of nasopharyngeal samples [17]. For inclusion in this study, a confirmed diagnosis of COVID-19, defined as a positive PCR result, was required.

The sample size was estimated to be 100 participants at a confidence level of 95% and test power of 80%. If any of the participants refused to give a sample, he/she was excluded from the study. This study was approved by the local ethics committee of Guilan University of Medical Sciences, Rasht, Iran (code: IR.GUMS.REC.1399.013). Written informed consent was obtained from each participant.

### Measurements

Nasopharyngeal, blood, urine, and stool samples were obtained from each participant at the time of admission, upon discharge, 1 week after discharge, and every 2 weeks until all samples were negative for SARS-CoV-2 RNA on RT-PCR. Upon admission, all four samples (nasopharyngeal, blood, urine, and stool) were collected from each participant and analyzed for SARS-CoV-2 RNA by PCR. Also, upon discharge, nasopharyngeal and stool samples were collected from each participant; blood and urine samples were collected if they were positive for SARS-CoV-2 RNA at the time of admission. Besides, in each follow-up visit, nasopharyngeal, blood, urine, and stool samples were collected if the patient was positive for SARS-CoV-2 RNA in the previous visit.

The nasopharyngeal and stool samples were obtained by sterile Dacron swabs. Also, 5-mL samples of whole blood and urine were taken for SARS-CoV-2-specific real-time RT-PCR. The samples were processed by a trained laboratory technician immediately after sampling. The coronavirus genome was isolated using the RNJia Virus Kit (ROJETechnologies, Yazd, Iran) for RNA extraction, and real-time PCR was carried out using the COVID-19 One-Step RT-PCR Kit (Pishtazteb, Iran), according to the manufacturer’s instructions. The results of quantitative RT-PCR are shown as cycle threshold (Ct) values. A positive control and a negative control were also included in each run to generate valid results. A Ct value < 40 was defined as a positive test result. Besides, the viral load was categorized as high (< 20), medium (20–29.9), and low (30–39.9) [18].

Moreover, the clinical and demographic characteristics of the participants were collected in this study. The sociodemographic characteristics of the patients included age, gender, marital status, job, education, type of residence, and socioeconomic status. Besides, the clinical manifestations of COVID-19 included fever, cough, sore throat, dyspnea, weakness, muscular pain, headache, diarrhea, nausea and vomiting, and chill. Besides, information on underlying diseases (e.g., diabetes, hypertension, cardiovascular disease, immunodeficiency, cancer, and respiratory disease) and inflammatory markers (e.g., white blood cell count, erythrocyte sedimentation rate, and C-reactive protein) were collected.

In this study, the hospital treatment plans were categorized into four groups, including hydroxychloroquine, antiviral treatment (e.g., lopinavir, Remdesivir, and Sovodak consisting of 400 mg sofosbuvir and 60 mg daclatasvir), interferon β1 (at five subcutaneous doses of 44 µg daily, 3 days a week), and local treatment (e.g., diphenhydramine, acetaminophen, zinc, vitamin C, and famotidine). All patients received corticosteroids (8 mg dexamethasone) daily.

### Data analysis

Comparison of qualitative variables (clinical and demographic characteristics) between the groups, categorized based on the duration of SARS-CoV-2 RNA persistence in the nasopharynx, was performed using Chi-square or Fisher’s exact test. A survival analysis was also performed to identify the median and 95th percentile of SARS-CoV-2 persistence. To find clinical and demographic characteristics that may be associated with the persistence of SARS-CoV-2 RNA, a Cox regression analysis was performed. Pearson’s correlation coefficient was also calculated to determine the relationship between the Ct value and the length of hospital stay. Data analysis was performed in SPSS version 17.0 (SPSS Inc., Chicago, IL, USA).

## Results

**Fig. 1:**
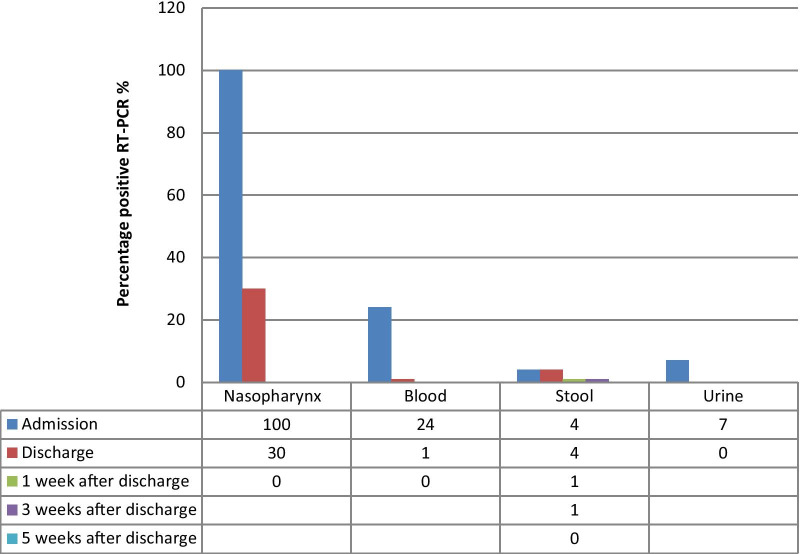
Result of SARS-CoV-2 RNA RT-PCR on different sample, over time (*A* admission, *D* discharge, *wk* week, *RT-PCR* reverse transcription-polymerase chain Reaction

During the study, 106 hospitalized patients with positive nasopharyngeal RT-PCR of SARS-CoV-2 RNA were enrolled, six of whom expired (5.66%). The mean age of the participants was 53.30 ± 13.03 years (range 29–86 years). The majority of the patients were male (60%), married (84%), urban residents (85%), and employed (68%). Also, the majority of the participants had less than high school diploma (63%) and a low socioeconomic status (82%). The results of SARS-CoV-2 RNA RT-PCR in different samples over time are shown in Fig. 1.

Only 24% of blood RT-PCR samples were positive upon admission; one sample remained positive upon discharge and became negative after 1 week. Also, the RT-PCR of urine samples was positive in 6% of the patients upon admission, while all samples became negative at discharge. Moreover, the RT-PCR result was positive in the stool samples of four patients upon admission, and two samples remained positive at discharge. Besides, two new stool samples were positive at discharge. Three of these samples were negative after 1 week, and one was negative 5 weeks after discharge. Upon discharge, 30% of nasopharyngeal RT-PCR samples were positive, and all were cleared after one weak.

There was no significant difference in terms of the demographic characteristics among the three groups. The participants’ clinical and demographic characteristics, based on the duration of viral persistence in the nasopharynx, are shown in Table 1. Almost 11% of patients showed viral persistence in the nasopharynx longer than 14 days from the first positive PCR. The demographic characteristics of the patients were not significantly different among the three groups.

**Table 1:** Patients clinical and demographic characteristics based on duration of SARS-CoV-2 RNA persistence in the nasopharynx

	All patient	Duration of SARS-CoV-2 RNA persistence in the nasopharynx			P*
		≤ 7 days after admission	7–13 days after admission	≥ 14 days after admission	
Count	100	46 (46%)	43 (43%)	11 (11%)	
Age, years					0.878
< 50	39	17 (43.6%)	18 (46.2%)	4 (10.3%)	
≥ 50	61	29 (47.5%)	25 (41%)	7 (11.5%)	
Gender					0.479
Male	60	27 (45%)	28 (46.7%)	5 (8.3%)	
Female	40	19 (47.5%)	15 (37.5%)	6 (15%)	
Marital status					0.730
Single	16	7 (43.8%)	8 (50%)	1 (6.2%)	
Married	84	39 (46.4%)	35 (41.7%)	10 (11.9%)	
Job					0.598
No	32	14 (43.8%)	13 (40.6%)	5 (15.6%)	
Yes	68	32 (47.1%)	30 (44.1%)	6 (8.8%)	
Education					0.441
Less than diploma	63	26 (41.3%)	29 (46%)	8 (12.7%)	
Diploma and more	37	20 (54.1%)	14 (37.8%)	3 (8.1%)	
Residency					0.195
Urban	85	40 (47.1%)	34 (40%)	11 (12.9%)	
Rural	15	6 (40%)	9 (60%)	0 (0%)	
Socioeconomic status					0.280
Low	82	37 (45.1%)	35 (42.7%)	10 (12.2%)	
Moderate to high	18	9 (50%)	8 (44.4%)	1 (0.06%)	
Underling disease					0.945
No	50	23 (46%)	21 (42%)	6 (12%)	
Yes	50	23 (46%)	22 (44%)	5 (10%)	
O_2_ saturation	93.84 ± 4.33	93.85 ± 4.03	93.88 ± 3.98	93.64 ± 6.80	0.986
Hospital treatment plan^a^					0.477
Hydroxychloroquine	5	2 (40%)	2 (40%)	1 (20%)	
Local treatment ^b^	62	32 (51.6%)	25 (40.3%)	5 (8.1%)	
Anti-viral ^c^	18	5 (27.8%)	11 (61.1%)	2 (11.1%)	
Interferon	15	7 (46.7%)	5 (33.3%)	3 (20%)	
*COVID-19 symptoms*					
Dyspnea					0.122
No	44	18 (40.9%)	18 (40.9%)	8 (18.2%)	
Yes	56	28 (50%)	25 (44.6%)	3 (5.4%)	
Sore throat					0.586
No	78	38 (48.7%)	32 (41%)	8 (10.3%)	
Yes	22	8 (36.4%)	11 (50%)	3 (13.6%)	
Muscular pain					0.152
No	82	37 (45.1%)	38 (46.3%)	7 (8.5%)	
Yes	18	9 (50%)	5 (27.8%)	4 (22.2%)	
Headache					0.470
No	76	34 (44.7%)	32 (42.1%)	10 (13.2%)	
Yes	24	12 (50%)	11 (45.8%)	1 (4.2%)	
Diarrhea					0.795
No	84	38 (45.2%)	36 (42.9%)	10 (11.9%)	
Yes	16	8 (50%)	7 (43.8%)	1 (6.2%)	
Fever					0.991
No	19	9 (47.4%)	8 (42.1%)	2 (10.5%)	
Yes	81	37 (45.7%)	35 (43.2%)	9 (11.1%)	
Cough					0.249
No	30	14 (46.7%)	15 (50%)	1 (3.3%)	
Yes	70	32 (45.7%)	28 (40%)	10 (14.3%)	
Weakness					0.550
No	93	43 (46.2%)	39 (41.9%)	11 (11.8%)	
Yes	7	3 (42.9%)	4 (57.1%)	0 (0%)	
Nausea and vomiting					0.145
No	79	40 (50.6%)	32 (40.5%)	7 (8.9%)	
Yes	21	6 (28.6%)	11 (52.4%)	4 (19%)	
Chills					0.886
No	21	9 (42.9%)	10 (47.6%)	2 (9.5%)	
Yes	79	37 (46.8%)	33 (41.8%)	9 (11.4%)	
Inflammatory markers					
WBC, cell/mL	7.41 ± 3.95	7.15 ± 3.42	7.96 ± 4.63	6.37 ± 3.05	0.415
ESR, mm/h	53.38 ± 28.11	50.78 ± 29.64	55.88 ± 26.42	54.45 ± 29.66	0.692
CRP, mg/L					
< 12	24	11 (23.9%)	11 (25.6%)	2 (18.2%)	0.473
12–20	16	6 (13%)	9 (20.9%)	4 (36.4%)	
> 20	57	29 (63%)	23 (53.5%)	5 (45.5%)	
Ct value(mean ± SD)	29.67 ± 4.69	29.98 ± 4.45	29.33 ± 5.25	29.73 ± 3.47	0.809
Nasopharyngeal viral load at admission, n (%)					0.464
High (Ct < 20)	1	0 (0%)	1 (100%)	0 (0%)	
Medium (Ct 20–29.9)	47	19 (40.4%)	22 (46.8%)	6 (12.8%)	
Low (Ct ≥ 30)	52	27 (51.9%)	20 (38.5%)	5 (9.6%)	

Continuous variables are presented as mean ± SD

*COVID-19* Coronavirus disease 2019, *SARS-CoV-2 RNA* sever acute respiratory syndrome coronavirous2, *WBC* Wight blood cells, *ESR* Erythrocyte sedimentation rate, *CRP* C-reactive protein, *Ct value* cycle threshold value

^*^Statistical significance based on the Chi-square or ANOVA test, Pearson correlation coefficient

^a^All of the patients received corticosteroids

^b^Including diphenhydramine, acetaminophen, zinc, vitamin C, famotidine

^c^Including lopinavir, Sovodak (Sofosbuvir at 400 mg and daclatasvir at 60 mg, Remdesivir)

Similarly, there was no significant association between the COVID-19 symptoms and the duration of viral persistence in the nasopharynx. Our findings revealed that the type of hospital treatment plan did not contribute to the viral persistence in the nasopharynx. In this study, only one out of 100 patients (1%) had a high viral load, 47% had a moderate viral load, and 52% had a low viral load. There was no significant relationship between the Ct value and the length of hospital stay (r = 0.030, *P* = 0.766). Also, no significant difference was detected between the duration of SARS-CoV-2 RNA persistence in the nasopharynx and the nasopharyngeal viral load upon admission (r = 0.030, *P* = 0.766).

**Fig. 2:**
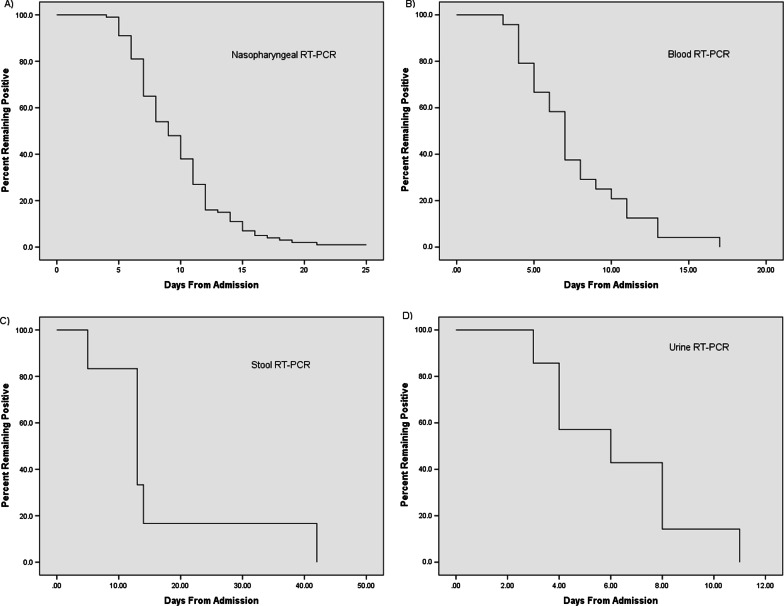
The duration for a positive SARS-CoV-2 RNA RT-PCR test to turn negative. **a** Nasopharyngeal RT-PCR (n = 100). **b** Blood RT-PCR (n = 24). **c** Stool RT-PCR test (n = 6). **d** Urine RT-PCR test (n = 7)

The median duration of SARS-CoV-2 RNA persistence in the nasopharynx was 8 days (95% CI 6.91–9.09) from the first positive RT-PCR upon admission, and the maximum duration of viral persistence was 25 days from admission (Fig. 2). The median duration of SARS-CoV-2 RNA persistence in the blood was 7 days (95% CI 6.07–7.93) from the first positive RT-PCR at admission, and the maximum duration of viral persistence was 17 days from admission (Fig. 2). The median duration of SARS-CoV-2 RNA persistence in the stool samples was 13 days (95% CI 6.96–19.4) from the first positive RT-PCR, and the maximum duration of viral persistence was 42 days from admission (Fig. 2). Besides, the median duration of SARS-CoV-2 RNA persistence in the urine was 6 days (95% CI 4.16–8.41) from the first positive RT-PCR upon admission, and the maximum duration of viral persistence was 11 days from admission (Fig. 2).

**Fig. 3:**
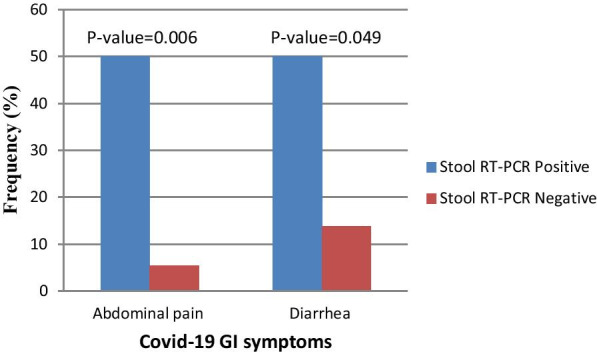
Frequency of COVID 19 gastrointestinal (GI) symptoms according to result of stool RT-PCR (Statistical significance based on Fisher's Exact test)

In the Cox regression analysis of factors possibly associated with the duration of SARS-CoV-2 RNA persistence, no significant associations were found (data not presented). The frequency of some COVID-19 gastrointestinal (GI) symptoms, according to the stool RT-PCR, is presented in Fig. 3. Abdominal pain and diarrhea were significantly more common in patients with positive stool RT-PCR samples as compared to those with negative stool RT-PCR. Moreover, in the analysis of clinical and demographic characteristics that could be associated with positive blood and urine RT-PCR, no significant relationships were detected (data not presented).

## Discussion

In this longitudinal study, the maximum duration of nasopharyngeal (25 days) and fecal (42 days) SARS-CoV-2 RNA shedding was longer than the recommended quarantine duration (at least 10 days after symptom onset and after resolution of fever for at least 24 h) [13]. Nearly one-third of hospitalized COVID-19 patients had positive nasopharyngeal RT-PCR results upon discharge, and about one-tenth of them showed viral persistence in the nasopharynx longer than 14 days from the first positive RT-PCR. The maximum duration of nasopharyngeal shedding was 25 days from admission.

The mentioned findings are compatible with the results of some studies, which revealed that some patients continued to have positive upper respiratory tract RT-PCR results after discharge from the hospital for the next few days [19, 20]. Although we did not identify any determinants of viral persistence, a study from China demonstrated that the prolonged presence of the virus in the upper respiratory tract was associated with disease severity [21]. On the other hand, another study from Portugal revealed that viral RNA persistence was not associated with disease severity, and a stronger immune response was a determinant of viral RNA clearance [22].

Positive blood SARS-CoV-2 RNA RT-PCR results were reported in about one-third of our patients, although most of them became negative at the time of discharge. Although studies on SARS-CoV-2 RNA detection in the blood are limited, a study from China revealed that SARS-CoV-2 RNA was detected in the blood of six out of 57 Chinese patients; all six patients with positive blood RT-PCR had a severe clinical condition [23]. The higher positive rate of blood RT-PCR in our study was probably attributed to disease severity in our patients and the hospital-based design of this study.

The present findings revealed that positive results of urine SARS-CoV-2 RNA RT-PCR were less common (found in 7/100 patients) in our study population, all of which became negative at the time of discharge. This result is consistent with previous studies from Turkey and China, which demonstrated that nearly 5–7% of COVID-19 patients had positive urine RT-PCR results [24, 25].

Positive stool SARS-CoV-2 RNA RT-PCR was detected in only six out of 100 patients in our study, while the time required for the positive stool RT-PCR to become negative was longer than the nasopharyngeal, urine, and blood RT-PCR tests, with a maximum fecal shedding duration of 42 days. Therefore, positive stool RT-PCR results in hospitalized patients represent the need to take precautions and use protective equipment in interventional procedures involving the gastrointestinal tract in a hospital environment. Similarly, in another study from China, an asymptomatic case remained positive in the stool RT-PCR for a period of 42 days. Also, in almost two-thirds of the patients, fecal shedding took longer than nasopharyngeal shedding [20]. Consistent with these findings, longer periods were reported in previous studies for detecting and shedding other coronaviruses in the gastrointestinal tract [26, 27].

According to our findings, the viral load of SARS-CoV-2, measured by the Ct method upon admission, is not a valuable parameter for predicting the duration of SARS-CoV-2 RNA clearance from the nasopharynx. The Ct value was inversely associated with the length of hospital stay and the time until viral clearance; in other words, a higher Ct value was indicative of a faster viral clearance [28, 29].

Although our findings demonstrated that the maximum duration of nasopharyngeal and fecal SARS-CoV-2 RNA shedding was longer than the recommended quarantine duration by the CDC [13], it is unclear whether individuals with persistent positive RNA PCR results have an infection risk. Therefore, further studies are needed to determine whether PCR positivity is associated with infective or non-infective nucleic acid fragments. In the present study, as soon as the patients were discharged from the hospital, there was no viral genetic material in the blood, urine, or nasopharyngeal samples, except in two out of 100 patients with a persistent PCR. Therefore, patients treated for SARS-CoV-2 and COVID-19 did not show infectivity once discharged from the hospital; this finding provides further evidence for applying successful treatments [30–32].

The strengths of the present study include the long-term follow-up and detection of viral RNA in respiratory and extra-respiratory sites. On the other hand, the hospital-based design of this study is one of its limitations, leading to a more strict patient enrollment that limited the generalizability of our findings. Another limitation of this study is that the day of symptom onset was not determined, and all calculations and analyses were based on the first nasopharyngeal RT-PCR at admission; therefore, the duration of viral shedding was underestimated. Finally, since SARS-CoV-2 RNA was found upon discharge in a reduced number of samples (especially in the blood and stool samples), further large-scale studies are recommended.

## Conclusion

The present findings revealed that in hospitalized COVID-19 patients, the maximum duration of nasopharyngeal and fecal SARS-CoV-2 RNA shedding was longer than the recommended quarantine duration by the CDC. However, immediately after the patients were discharged from the hospital, there was no evidence of viral genetic materials; therefore, appropriate treatments were applied in this hospital. However, further, multi-center and prospective randomized controlled trials are recommended on a larger sample size to evaluate the effects of different drugs on viral shedding through body secretions.

## Data Availability

The datasets obtained during this study will be available upon request to the corresponding author.

## Abbreviations

CDC: the centers for disease control and prevention
RT-PCR: real-time fluorescent quantitative polymerase chain reaction
